# Prenatal Stress and Ethanol Exposure: Microbiota-Induced Immune Dysregulation and Psychiatric Risks

**DOI:** 10.3390/ijms25189776

**Published:** 2024-09-10

**Authors:** Rosana Camarini, Priscila Marianno, Maylin Hanampa-Maquera, Samuel dos Santos Oliveira, Niels Olsen Saraiva Câmara

**Affiliations:** 1 Department of Pharmacology, Institute of Biomedical Sciences, Universidade de São Paulo, São Paulo 05508-900, Brazil; primarianno@usp.br (P.M.); maylin.hanampa@usp.br (M.H.-M.); 2 Department of Immunology, Institute of Biomedical Sciences, Universidade de São Paulo, São Paulo 05508-900, Brazil; oliveirass@usp.br

**Keywords:** microbiota, inflammation, immune system, depression, anxiety

## Abstract

Changes in maternal gut microbiota due to stress and/or ethanol exposure can have lasting effects on offspring’s health, particularly regarding immunity, inflammation response, and susceptibility to psychiatric disorders. The literature search for this review was conducted using PubMed and Scopus, employing keywords and phrases related to maternal stress, ethanol exposure, gut microbiota, microbiome, gut–brain axis, diet, dysbiosis, progesterone, placenta, prenatal development, immunity, inflammation, and depression to identify relevant studies in both preclinical and human research. Only a limited number of reviews were included to support the arguments. The search encompassed studies from the 1990s to the present. This review begins by exploring the role of microbiota in modulating host health and disease. It then examines how disturbances in maternal microbiota can affect the offspring’s immune system. The analysis continues by investigating the interplay between stress and dysbiosis, focusing on how prenatal maternal stress influences both maternal and offspring microbiota and its implications for susceptibility to depression. The review also considers the impact of ethanol consumption on gut dysbiosis, with an emphasis on the effects of prenatal ethanol exposure on both maternal and offspring microbiota. Finally, it is suggested that maternal gut microbiota dysbiosis may be significantly exacerbated by the combined effects of stress and ethanol exposure, leading to immune system dysfunction and chronic inflammation, which could increase the risk of depression in the offspring. These interactions underscore the potential for novel mental health interventions that address the gut–brain axis, especially in relation to maternal and offspring health.

## 1. Introduction

The influence of maternal stress and alcohol exposure on the gut microbiome presents a compelling avenue of research with implications for the well-being of both mothers and their offspring. Although human studies in this field remain limited, emerging preclinical evidence suggests that changes in maternal gut microbiota due to stress and/or alcohol exposure can have lasting effects on offspring’s health. Understanding the dynamic interactions among stress, depression, and microbiota holds promise for developing innovative mental health interventions that consider the role of the gut–brain axis. Throughout this review, the terms “ethanol” and “alcohol” are used interchangeably to refer to the same substance—drinking alcohol.

The gut microbiota can communicate bidirectionally with the central nervous system (CNS) through the gut–brain axis [1], impacting the quality of life and the risk of depression [2]. Chronic stress alters the composition and diversity of the gut microbiota, potentially influencing mental health [3]. Dysregulation of this fine-tuned interaction may contribute to the onset or exacerbation of depression.

The early stages of life are crucial for immune system development, and the composition of the gut microbiota during this period plays a pivotal role in shaping the immune system itself and its responses. Stress affects maternal physiology by triggering the release of stress hormones like cortisol, which in turn impacts various systems, including the gut microbiota [4]. In parallel, prenatal ethanol exposure is recognized for its adverse effects on maternal health, and for the heightened risk of inducing Fetal Alcohol Spectrum Disorders [5]. Some of the negative effects of ethanol include disruption in physiological processes such as nutrient absorption, digestion, and metabolic pathways linked to gut microbiota composition [6].

Stress and ethanol consumption during pregnancy often interact in a reciprocal dynamic, wherein maternal stress can serve as a trigger for ethanol use as a coping mechanism [7], while ethanol, in turn, can exacerbate stress-related responses [8]. Studies have reported that some women consume alcohol during pregnancy because they believe it reduces stress, aids sleep, and acts as a relaxant to ease labor and delivery. They also perceive alcohol to be less harmful than other substances [9,10]. This mutual relationship underscores the rationale for this review due to its profound impact on maternal health and potential implications for offspring’s gut microbiota and immunity.

The amount of ethanol necessary to induce dysbiosis can vary depending on several factors, including the duration of ethanol exposure and the specific composition of the diet. Acute ethanol exposure can also lead to dysbiosis in mice [11], although the effects are generally more pronounced and sustained with chronic exposure in both rats and humans [12]. Similarly, both single [13] and repeated stress [14] can alter gut bacterial composition in rodents.

During pregnancy, exposure to ethanol and stress can have particularly severe consequences. The combination of ethanol and stress can disrupt the maternal gut microbiota balance, potentially leading to altered fetal development, impaired immune system function in the offspring, increased susceptibility to immune-related disorders, allergies, autoimmune diseases, and a heightened risk of neurodevelopmental disorders [15,16]. Furthermore, maternal microbiota changes may set the stage for long-term microbiota dysbiosis in the child, potentially linked to inflammation, a contributing factor in depression [17].

This review aims to synthesize preclinical and clinical research on the effects of stress and ethanol on maternal gut microbiota, elucidating their potential transgenerational impacts on offspring’s immunity, inflammation response, and susceptibility to depression. Moreover, this approach offers a more nuanced view that these elements may interact and influence maternal and fetal health.

## 2. Microbiota Host: From Passive Spectator to Essential Modulator in Health and Disease

### 2.1. Structural Components of Microbiota Host

Recent studies have revolutionized our understanding of microbiota–host interactions, revealing that the intestinal microbiota is not merely a passive observer but an important component in modulating the host organism’s homeostasis, directly influencing health and disease [18]. This interaction between commensal microorganisms and humans has occurred since the emergence of our species on the planet. It was evolutionarily selected in our distant ancestors due to the significant benefits it provided to both parties: the host offered shelter and nutrients, while the microorganisms aided in the degradation of dietary fibers and occupied niches formed by epithelial barriers, thereby preventing or hindering colonization by pathogenic microorganisms [19].

The biological entity constituted by the host and all the microorganisms that colonize it is called holobiont, and the intimate connection between its components allows for the expansion of individual physiological capabilities [19]. A classic example is the degradation and fermentation of food components, conducted by intestinal bacteria, to form short-chain fatty acids, which are molecules that are extremely important for human health [20]. A wide range of host biological processes can be modulated by the microbiota and its secreted components. The impact of the microbiota on the modulation of innate and adaptive immunity [21], metabolism [22], circadian cycle [23], and behavior [24] is well-established. Conversely, its dysregulation is associated with various disorders, such as autoimmunity [25], metabolic syndrome [26], neurodegenerative diseases [27], and psychiatric diseases [28].

The microorganisms that constitute the intestinal microbiota with a beneficial function for the host, in essence, are similar to pathogenic microorganisms. However, our immune system uses several devices to identify whether the presence of a microorganism represents a danger or not. The structure of the intestinal barrier is specifically designed to physically separate the microbial compartment (the intestinal lumen) from the immune cell compartment, primarily located in the lamina propria, Peyer’s patches, and mesenteric lymph nodes [19].

This intestinal barrier operates through various mechanisms: physical, chemical, immunological, and microbiological barriers. Regarding physical barriers, intestinal epithelial cells are organized in juxtaposition and tightly adhered to one another thanks to tight junction proteins such as occludin, claudins, and zonula occludens. This structure endows the intestine with highly selective permeability, allowing essential molecules to pass while restricting the translocation of microorganisms to the lamina propria, a process that commonly occurs after intestinal injuries [29] or changes in intestinal permeability, which will be discussed later in this review. Adjacent to the intestinal epithelium lies the mucus layer, a gel-like, viscous, elastic, and highly hydrated matrix with Mucin 2 (MUC2) as its fundamental structural component. MUC2 molecules are secreted by specialized intestinal epithelial cells called goblet cells and interact with each other via disulfide bridges, as well as with other gel-forming mucins such as MUC6, MU19, MUC5B, and MUC5AC [30]. This layer not only physically separates bacteria from the host’s tissues but also hydrates, lubricates, and protects the intestinal lumen [31].

The mucus layer also serves as a chemical barrier due to its ability to directly bind to bacterial peptidoglycans and control their growth [32]. However, another class of peptides known as antimicrobial peptides are the classic components of the chemical barrier. In mammals, C-lectins, defensins, histidines, and cathelicidins are key components in antibacterial defense, being prominently produced by neutrophils and epithelial cells, not only in the intestine but also in other barrier tissues [33]. In the intestine, Paneth cells are specialized epithelial cells responsible for the production of antimicrobial peptides, located in the crypts of the small intestine where they maintain close contact with intestinal stem cells [34].

Another class of specialized epithelial cells is M cells. They are cells devoid of glycocalyx components in their membrane, unlike what is observed in other cells of the intestinal epithelial monolayer. In these cells, the glycocalyx results in microfolds, which justifies its name. These cells connect the mucous and microbiological layer with the immunological layer, and their folds are essential in this process as they help in the capture of antigens and microorganisms present in the lumen and transport them through endocytic vesicles for delivery to dendritic cells (DCs) in the Peyer’s patches [35]. This antigenic sampling can also be conducted directly by the DCs underlying the intestinal epithelium. They are able to project their dendrites between the enterocytes and capture antigens, all without compromising the structure and permeability of the intestinal epithelium since these DCs also express tight junction proteins. Thus, there is a rapid loosening of these junctions between enterocytes, followed by re-stabilization with the junction proteins present in DCs [36,37].

### 2.2. Immunological Components of Microbiota Host

After capturing antigens, directly from the lumens or via M cells, DCs migrate to the mesenteric lymph nodes (mesLN) where they activate T cells specific to that antigen, promoting their proliferation and imprinting on these cells, which are important characteristics to guide their migratory profile. In this process, activated DCs in the gut release high levels of retinoic acid, a component formed from Vitamin A from food, and induce the expression of chemosign receptors α4β7 and C-C motif chemokine receptor type 9 (CCR9) in T cells, which are responsible for the migration of these cells to the lamina propria [38]. In an infectious context, these intestinal T cells have a lower rate of cytokine proliferation, but with longer activity, which ensures a local and direct response to the microorganism, the damage to the intestinal barrier is minimal [39,40].

Among helper T cells (Th), regulatory T cells (Treg) and Th17 are the most abundant and play a central role in maintaining the intestinal barrier, the first providing tolerance to microbiota and food antigens [41,42], and the second promoting resistance mainly to fungi and bacteria that may cause infection [43,44]. Both cell populations require the presence of transforming growth factor beta in their differentiation process, a cytokine widely present in the intestine. Even under homeostatic conditions, the presence of retinoic acid also helps activated T cells in the gut to differentiate into Treg while inhibiting their differentiation into Th17 cells [45]. However, the presence of interleukin (IL)-6, a pro-inflammatory cytokine rapidly produced in response to various infections, together with transforming growth factor beta, polarizes T cells towards the Th17 profile [46].

The immune system and commensal microorganisms directly interact, influencing each other in both health and disease. On one hand, the intestinal microbiota helps train immune cells in homeostasis, preparing them to detect pathogens [47]. Meanwhile, the immune system maintains the balance of the relationship by tolerating the microbiota while also “pruning” its excessive growth. In immunocompromised individuals, commensal microorganisms can proliferate uncontrollably, leading to infections. Conversely, overly aggressive immune responses against the microbiota can damage the host’s tissues, leaving their niche vulnerable to colonization by pathogenic microorganisms [18].

Extrinsic factors, such as eating habits, can also affect the host’s microbiota. Recent studies point to the ability of high-sugar diets to deplete segmented filamentous bacteria, a class of bacteria crucial for inducing Th17 cells in the intestine. Sugar, together with IL-22 produced by Type 3 innate lymphoid cells (ILC3), promotes the growth of *Faecalibaculum rodentium* and the elimination of segmented filamentous bacteria. In this context, Th1 cells that produce interferon-gama (IFNγ) begin to predominate in the lamina propria, promoting intestinal inflammation and increased permeability, which allows the translocation of bacteria to the liver, intensifying the inflammatory process and contributing to insulin resistance, a key factor in the development of metabolic syndrome [18]. The constitutional change of the microbiota in response to dietary changes occurs rapidly, with a switch from a fiber-rich diet to a sugar-rich diet for just 3 days being sufficient to alter the functional profile of the microbial community [48]. Moreover, alterations in the microbiota and sugar levels can “deprogram” T lymphocytes, rendering them temporarily unresponsive and leaving the host more susceptible to infections [49].

Dysbiosis is considered an altered state of the intestinal bacterial community. Although this definition appears simple, there are a series of complexities regarding what would be a normal microbiota pattern [50], especially when considering its great taxonomic diversity due to environmental, nutritional, and genetic factors. As a result, the definition of normality can vary over time and across different geographical locations [51,52].

Dysbiosis can result from factors like stress and alcohol exposure, as well as diet, antibiotics, age, and inflammation, among others, potentially leading to brain dysfunction due to the gut–brain connection. Bacterial extracellular vesicles have been identified as potential mediators of this communication by interacting with immune receptors and triggering neuroinflammatory responses. These extracellular vesicles, containing harmful substances such as lipopolysaccharides (LPS) and toxins, can cross barriers like the blood–brain barrier or placental barrier and activate immune receptors, such as toll-like receptors (TLRs) on glial cells, resulting in cytokine and inflammatory mediator production that can impair brain function and behavior [53].

## 3. Maternal Microbiota Disturbance: Implications for Offspring Immune System and Beyond

### 3.1. Maternal DIET and Microbiota

Abrupt changes in diet during pregnancy, combined with fluctuations in hormonal levels—especially increased production of progesterone and estrogens—are associated with weight gain, elevated blood glucose levels, even during fasting, and insulin resistance [54,55]. Adequate maternal intake of macronutrients and micronutrients is crucial for the proper development of the fetal immune system. Essential macronutrients include carbohydrates, lipids, and proteins, while critical micronutrients comprise minerals such as zinc, selenium, copper, iodine, and iron, along with vitamins like A, C, D, E, and folate. Inadequate maternal nutrition during pregnancy, particularly protein–energy malnutrition, can lead to dysfunction in the mother immune cells [56]. Moreover, maternal diet influences the development of both the adaptive and innate immune systems in the offspring. For example, maternal intake of certain fats can modulate the risk of developing conditions like eczema and allergies in children, which are related to the adaptive immune response. The quality and composition of maternal diet fats (saturated vs. polyunsaturated, N-3 vs. N-6 polyunsaturated fatty acids) are important for modulating the immune response. Particularly, the intake of N-3 can lead to lasting epigenetic changes in the offspring’s immune cells, affecting their immune responses [57,58].

Non-human studies have shown the effects of a high-fat diet on the offspring’s microbiome. Female macaques fed with a high-fat diet (36% fat) had distinct microbiota compared to the controls (13% fat). The offspring of mothers fed a high-fat diet exhibited an altered gut microbiome that persisted even after being weaned onto a control diet, showing a lasting reduction in *Campylobacter* [58]. A high-fat diet is associated with an increase in Gram-negative bacteria, a higher Firmicutes:Bacteroidetes ratio, colonic inflammation, and a decrease in regulatory T cells. These effects were passed to the offspring of mice fed with a Western diet (40–60% fat, primarily saturated) but reversed by altering the microbiota, suggesting that microbiota changes are the main drivers of diet-induced immune effects. Western diet pups had a higher abundance of the Lachnospiraceae and Clostridiales compared to the low-fat pups [59].

### 3.2. Influence of Progesterone on Microbiota during Pregnancy

**Figure 1:**
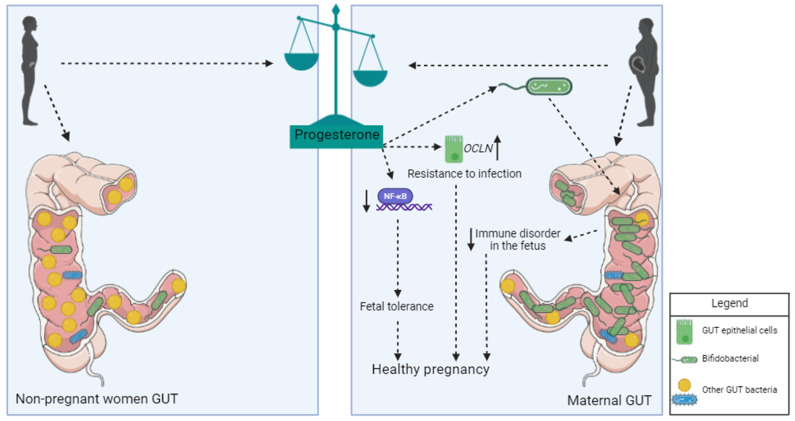
Modulation of intestinal microbiota, immunity, and progesterone levels during pregnancy. During pregnancy, elevated progesterone levels act on intestinal epithelial cells, increasing the expression of occludins to enhance resistance to infections. These changes reduce NF-KB activation in immune cells to tolerate the fetus and promote the growth of Bacteroides, microorganisms that are passed to the fetus and provide protection against immunopathologies. ↑ = upregulated; ↓ = downregulated.

Progesterone plays an important role in controlling the microbiota of pregnant women and the physical barriers of the intestine (Figure 1). Studies report that the hormone acts on the epithelial cells of the intestinal barrier, promoting increased expression of occludin and, therefore, strengthening tissue impermeability and reducing the likelihood of intestinal infections [60]. Additionally, the hormone acts directly on beneficial bacteria during pregnancy, promoting their expansion. Bacteria of the genus *Bifdobacterium* have the enzyme hydroxysteroid dehydrogenase in their membrane, which has the ability to metabolize progesterone [61]. Progesterone levels in feces negatively correlate with the concentration of LPS in the blood of pregnant women, a strong indication of the role of this hormone in regulating the intestinal microbiome and protecting the host against infections. The host response to bacterial LPS is also regulated by progesterone, which inhibits the activation of nuclear factor kappa B (NF-KB) and reduces the exacerbated inflammatory response, a risk factor for pre-eclampsia and premature birth [62].

### 3.3. Maternal Microbiota and Immunity: Implications for Fetal and Neonatal Health

The effector functions of the immune system that promote tolerance or resistance are spatially and temporally separated during pregnancy. Immunosuppression in the placental barrier, which is important to ensure that the fetus is not rejected, is not observed in the mucous membranes. In fact, mothers have very efficient immunological responses in these areas, which is extremely important due to the serious risks that infections during pregnancy pose to both mother and baby [62]. Furthermore, the regulatory immune responses in the placental barrier are particularly strong during the 3rd month of pregnancy and gradually decline as birth approaches [60]. The amniotic fluid also helps to regulate maternal immunity and influences immune responsiveness during the fetal and neonatal periods [63,64]. The immunomodulatory properties of amniotic fluid have been attributed to alpha-fetoprotein in regulating natural killer cells and T cell-mediated immune reactions [20]. However, Lang et al. have demonstrated that the primary mediators are not alpha-fetoprotein, but rather the transforming growth factors TGF-β1 and β2 [65].

The microbiota lives in close contact with individuals throughout their lives, but the existence of microbiota in amniotic fluid remains controversial. While it appears that the fluid is not a source of microorganisms for in utero colonization in mice, it may still contribute to fetal exposure to microbial components [66]. Studies have detected microbial DNA in human placental tissues and fluids [67]. However, it remains unclear whether this DNA indicates the presence of viable local organisms from past colonization or is the result of translocation from the bloodstream. Viable bacteria are scarce in the fetal intestine at mid-gestation, but specific strains with immunomodulatory potential, such as Micrococcus luteus, have been identified and may influence fetal immune development [68]. Evidence suggests that paternal microorganisms from semen can contribute to the uterine microbiome, potentially by transporting cervical bacteria upstream during fertilization [69,70]. It is well known that the majority of babies’ microbiota originates from the mother’s microbiota and the surrounding environment [71,72,73]. Thus, maintaining a balanced maternal microbiota is crucial for the initial constitution of newborns’ microbiota. Commensal microorganisms are especially important because they protect newborns, who are highly susceptible to infections due to their immature immune systems [74].

The fetal microbiota also plays a role in regulating immune responses. Pro-inflammatory cytokine production is reduced in human T cells stimulated with live *Micrococcus luteus*, the most prevalent bacterium in the fetal intestine, from viable fetuses. This suggests that the fetal microbiota helps generate immunological tolerance. *M. luteus* stimulates the development of dendritic cells and T cells in the fetal intestine [68]. While it is unclear whether these cells have a tolerogenic profile, they likely help control intestinal microbial populations and tolerate their presence, possibly through simpler mechanisms than those observed in adults, such as the production of IL-10 and TGF-β.

In addition to protecting against infections, the neonatal microbiota is crucial for protection against metabolic diseases like obesity. High levels of bifidobacteria in babies’ feces correlate with normal weight, while the abrupt presence of *Staphylococcus aureus* is associated with increased weight gain [75]. Bifidobacteria also protect against the development of allergies in neonates, while high levels of clostridia are linked to changes in the composition of short-chain fatty acids produced in the intestines of babies, making them more prone to allergies [76]. Colic, a common issue among babies, is also associated with microbiota imbalances. High levels of lactobacilli are found in babies without colic, while the presence of Gram-negative bacteria is more prevalent in babies with colic [77].

Hormonal, immunological, behavioral, and dietary changes during pregnancy affect the microbial composition of all barriers, starting from the 3rd month of pregnancy. In the intestine, there is an increase in Actinobacteria, Proteobacteria, and *Bifidobacterium*, in addition to a reduction in *Faecalibacterium* [78,79]. As previously discussed, the immune system shapes the diversity of the microbiome, and the microbiome modulates and trains the immune system. This is supported by observations showing that reduced intestinal colonization by bacteria from the *Faecalibacterium* genus is associated with increased local inflammation due to decreased production of butyrate, an important immunoregulatory agent produced by these bacteria [80].

The intimate interaction facilitated by the evolution of viviparity in mammals allows the transfer of various compounds produced by the mother and components of her microbiota to the fetus through the placenta. Viviparity emerged independently in different groups of living organisms with the common objectives of promoting nutrition, protection, and gas exchange to offspring [81]. In mammals, this relationship reaches its highest complexity, as evidenced by the existence of the placenta, an organ that allows the exchange of molecules between mother and fetus, while also functioning as a barrier to ensure a unique microenvironment for the fetus [82]. This process is essential for fetal development, and birth and plays a significant role in imprinting characteristics that affect the health of the offspring well beyond birth [83].

Using nonhuman primate models, Nash et al. (2023) [84] demonstrated that a maternal Western-style diet, rich in sugars and lipids, leads to the reprogramming of fetal hematopoietic stem and progenitor cells, causing a proinflammatory phenotype in the offspring. Their bone-marrow-derive macrophages are enriched in genes associated with NF-ΚB, as well as pathways associated with glycolytic metabolism, a profile that is maintained even after 3 years of birth. Similar results were found in macrophages differentiated from hematopoietic pluripotent cells, indicating that the mother’s diet induces epigenetic changes in hematopoietic progenitors that are maintained in terminally differentiated cells [84].

The immune system of newborns is strongly influenced by the intrauterine environment. Through the placenta, not only nutrients and oxygen are exchanged between mother and child, but also cells, microorganisms, and their metabolites are transferred from mother to baby [85]. This transfer, together with immunoglobulin (Ig) A molecules, also occurs during breastfeeding and is essential for the development of the intestinal barrier. Among all immunoglobulin isotypes in colostrum and milk, secretory IgA is the most important due to its high concentration and crucial role in defending mucous membranes, preventing microorganism entry into tissues and its anti-inflammatory properties [86]. Studying the impact of the maternal microbiota on the development of the fetal immune system is challenging, even in mice, because germ-free mothers birth germ-free babies, and colonized mothers deliver colonized babies. Therefore, determining whether changes in the development of children’s immune systems depend on their microbiota (or lack thereof) or on the maternal microbiota (or lack thereof) is a significant challenge. A model was developed to solve this equation, where pregnant germ-free females were colonized with a mutant strain of *Escherichia coli*, incapable of replicating in the human intestine due to a deficiency in synthesizing essential bacterial amino acids caused by mutations in genes associated with D-alanine biosynthesis. In this case, bacteria temporarily colonized the maternal intestine and were eliminated even before birth, resulting in germ-free offspring from mothers colonized only during pregnancy. This approach allowed for a direct demonstration of the positive impact of maternal microorganisms on the induction of Type 3 innate lymphoid cells and F4/80+CD11c+ mononuclear cells in the newborn intestine [87].

Direct microbial metabolites, or those resulting from the degradation of dietary fibers by intestinal commensals, are transferred to the offspring through the placenta and breastfeeding. These metabolites promote the expansion of immune cells in the intestine, contribute to the physical structuring of the intestinal barrier by stimulating the proliferation of epithelial cells, and induce the production and secretion of mucus, immunoglobulins, and defensins into the intestinal lumen [88]. Additionally, these compounds can act on hematopoietic progenitors present in the bone marrow of newborns, creating an inflammatory environment that imprints an inflammatory program on these cells, which may persist for years after birth in the offspring [89].

The relationship between the microbiota and the host extends beyond the gut, influencing local physiological processes and reaching the brain through the gut–brain axis [90]. This bidirectional communication system underscores the importance of maintaining a balanced microbiota for overall health [91], as dysregulation in the gut may contribute to neurological and psychiatric disorders [92,93].

## 4. Exploring the Interplay between Prenatal Maternal Stress, Microbiota, and Depression

### 4.1. Stress, Gut Microbiota, and Depression

This section explores the interaction among stress, gut microbiota, inflammation, and depression via the gut–brain axis. Chronic stress is a recognized risk factor for depression in humans [94] and non-human animals [95] and contributes to functional gastrointestinal disorders [96]. Drossman’s biopsychosocial model emphasizes the role of stress in gastrointestinal disorders, such as gastroesophageal reflux disease and Crohn’s disease [97]. In turn, alterations in the gut microbiota contribute to various physiological aspects, including inflammation and alterations in mood [2,98]. The bidirectional communication between the CNS and the gastrointestinal tract, where stressors affect the gut microbiota [99], may influence brain function and emotions. In fact, in the last decade, the “gut–brain axis” model has emerged as a promising therapeutic approach for stress-related disorders.

Chronic stress-induced gut dysbiosis is recognized as a significant factor in the development of depression, fostering inflammation and immune dysregulation through the induction of a “leaky gut syndrome” [98]. Inflammation plays a pivotal role in depression, evidenced by heightened levels of inflammatory cytokines and the activation of the nucleotide oligomerization domain (NOD)-like receptor family, pyrin domain-containing protein 3 (NLRP3) inflammasome in patients [100] and animal models [101]. The association between chronic stress and gastrointestinal diseases highlights the connection between depression and inflammatory conditions, such as inflammatory bowel diseases, involving mechanisms such as heightened levels of pro-inflammatory cytokines, alterations in vagal nerve signaling, gut dysbiosis, and changes in brain morphology [102]. 

Exposure to stress not only induces changes in gastrointestinal motility, secretion, and mucosal blood flow [103] but also disrupts the composition of intestinal microbiota [1] through mechanisms that involve stress hormones. Stress triggers the activation of the hypothalamic–pituitary–adrenal (HPA) axis, releasing a corticosterone-releasing factor from the hypothalamus, stimulating the release of adrenocorticotropic hormone and glucocorticoid hormones into the blood, such as cortisol in humans and corticosterone in rodents. These hormones bind to mineralocorticoid and glucocorticoid receptors of the epithelial cells of the gut, impacting intestinal barrier integrity and gut microbiota composition while increasing gut permeability [104,105]. Studies in germ-free mice have provided insights into the influence of microbiota on HPA axis development and stress responses, revealing that sterile environments lead to increased HPA axis activity, a phenomenon normalized by colonization with commensal bacteria [106].

As already mentioned in Section 2, the integrity of tight junction proteins, such as occludin and zonula occludens-1, is vital for maintaining intestinal and blood–brain barriers. Stress can compromise these barriers, by decreasing intestinal epithelial tight junction proteins (zonula occludens-1, occludin, claudin 1), increasing colon permeability and allowing harmful substances to enter the bloodstream and the brain, thereby increasing inflammation [29,107]. The elevated cortisol, a common outcome of stress, disrupts the integrity of the intestinal barrier, resulting in a “leaky gut” condition and an increase in LPS levels, known as endotoxemia, ultimately contributing to gastrointestinal distress [108]. The “leaky gut” phenomenon allows harmful substances like endotoxins to enter the bloodstream, causing chronic systemic and central inflammation, which hyperactivates the HPA axis, leading to heightened cortisol secretion. Depression, a stress-related mood disorder, is associated with imbalances in the HPA axis, with the gut microbiota playing a crucial role in modulating these disruptions [109,110].

Cortisol interacts with various immune cells through the widespread presence of glucocorticoid receptors, including those in the colonic epithelium [111]. This interaction has been implicated in shifts in colonic motility, potentially resulting in increased colonic transit and changes in the microbiota composition. Patients experiencing depression often exhibit elevated cortisol levels, accompanied by an increase in inflammatory responses, suggesting this can be one of the mechanisms by which depression may alter the gut microbiota [112].

Thus, the capacity of stress-induced alterations in microbiota to compromise the gut barrier function, potentially resulting in systemic inflammation and oxidative stress, introduces critical factors that contribute to susceptibility to a range of neuropsychiatric disorders, including depression [112]. It is essential to emphasize that the role of the microbiota–inflammasome axis in depression is highly intricate and should be considered as a multidirectional model. A definitive cause–effect relationship has not yet been clearly defined. A hypothesis proposes that inflammasome, a multiprotein complex that participates in the regulation of the immune system, is the central mediator through which stress contributes to depression [38], i.e., chronic inflammation can contribute to neural disruptions underlying depressive states. This hypothesis is supported by a number of studies. Elevated inflammation signals the brain to induce symptoms resembling depression, such as negative mood, fatigue, increased pain sensitivity, and cognitive deficits. Inflammatory mediators can also trigger clinical depression, particularly in vulnerable individuals. Antidepressant medications are less effective in patients with high levels of plasma inflammatory markers, inflammation-related gene variations, and proinflammatory gene-expression profiles [101]. In patients with major depression or in animal models of stress-induced depression, levels of pro-inflammatory cytokines in the blood, such as interleukin IL-1b, IL-6, IL-18, IL-1Ra, tumor necrosis factor-α (TNF-α), and inflammasome components in immune cells, such as NLRP3, are significantly upregulated [38,90,113]. Elevated levels of caspase-1, an apoptosis-associated speck-like protein containing a caspase recruitment domain (ASC) and a C-reactive protein have been observed in human patients with depression [113]. Furthermore, depression-like behaviors can be alleviated by inhibiting the NLRP3 gene or with antidepressant treatment. In addition, antidepressant drugs have been found to inhibit inflammasome activation, particularly the NLRP3, leading to a reduction in serum cytokine levels in mice [114]. NLRP3 is activated in response to cellular stress and infection, leading to the production of IL-1β, which is a pro-inflammatory cytokine responsible for the inflammation occurring in patients with inflammatory bowel disease [115]. In the intestines, the NLRP3 inflammasome modulates the intestinal microbiota [116], while the commensal microbiota stimulates NLRP3-dependent IL-1β release after intestinal injury, promoting inflammation [117]. The activation of intestinal inflammasome can affect the CNS through the vagus nerve, influencing feeding behaviors. Certain microbial metabolites can impact inflammasome activation in the gut, contributing to the balance between host-microbial mutualism and affecting the host’s metabolism and immune response. These data indicate that the activation of intestinal inflammasomes by the microbiota can lead to the production of molecules that affect the CNS via the vagus nerve, suggesting an essential innate immune pathway connecting the gut and the brain [90]. Collectively, these findings indicate that the gut microbiota plays a causal role in depression.

The reciprocal interaction between the NLRP3 inflammasome and the gut microbiota is substantiated by investigations with chronic unpredictable mild stress (CUMS). CUMS induced elevated levels of NLRP3, caspase-1, and the inflammasome adapter apoptosis-associated speck-like protein containing a caspase recruitment domain (ASC). Conversely, probiotics, known for their ability to shape the microbiota, mitigated the expression of the NLRP3 inflammasome. Particularly noteworthy, fecal transplantation from rats exposed to CUMS triggered NLRP3 inflammasome activation in recipient rats. The alterations in tight junction proteins and microbiota composition observed in the recipient rats closely mirrored those of the donor rats, providing compelling evidence for a link between the microbiota and NLRP3 inflammasome dynamics [118].

The absence of caspase-1, a component of the inflammasome complex, reduced depressive-like behaviors in mice under conditions of chronic restraint stress. This effect was paralleled by changes in gut microbiota composition induced by antibiotic treatment, resembling alterations observed in caspase-1-deficient mice [119]. 

In the context of diverse gut microbiota, it is plausible that specific pathobionts act synergistically with other factors to induce depression. Furthermore, Lowe et al. (2018) [120] demonstrated that changes in microbiota composition induced by antibiotics, which are known to decrease the gut microbiome load, mitigated inflammation in both the hippocampus and small intestine induced by alcohol exposure in mice, emphasizing the bidirectional relationship between the gut microbiota and brain.

Despite variations in methodological approaches, several studies conducted in both humans and animals have shown differences in the gut microbiota composition between healthy individuals and those expressing symptoms of depression. The most prominent link is associated with the Firmicutes:Bacteroidetes ratio. Rodents with higher levels of Bacteroidetes (e.g., families of Bacteroidaceae, Prevotellaceae, Rikenellaceae, and Porphyromonadaceae) and reduced Firmicutes (e.g., *Roseburia, Eubacterium, Clostridium, Lactobacillus*, and *Ruminococcus*) in their gut microbiota tended to exhibit depressive-like behavior [121]. Rodents with depressive-like behaviors and cognitive impairment due to antibiotic-induced microbiota depletion exhibit shifts in the abundance of beneficial bacteria, such as Bacteroides and Firmicutes, compared to their healthy counterparts [122]. Individuals with major depressive disorder (MDD) exhibit an imbalance in gut microbiota, such as increased Enterobacteriaceae and *Alistipes* and reduced *Faecalibacterium* [123], while probiotic administration (*Lactobacillus acidophilus*, *Lactobacillus casei*, and *Bifidobacterium bifidum*) significantly reduces depressive symptoms in MDD patients [124]. A meta-analysis revealed that patients with MDD have lower quantities of bacteria from the Veillonellaceae, Prevotellaceae, and Sutterellaceae families but higher levels of Actinomycetaceae when compared to healthy controls. Furthermore, MDD patients exhibit decreased levels of specific genera like *Coprococcus, Faecalibacterium, Ruminococcus, Bifidobacterium*, and *Escherichia*, along with increased levels of *Paraprevotella* [125]. 

Regarding bacterial α-diversity in MDD patients, some studies noted reduced bacterial diversity [92,126], while Jiang et al. found increased levels of fecal bacterial α-diversity in patients diagnosed with clinically significant depression, such as Bacteroidetes, Proteobacteria, and Actinobacteria [123]. Additionally, assessments of β-diversity, with the aim of discerning differences in the overall microbial community composition between healthy individuals and those with MDD, effectively distinguished patients from healthy controls [127]. It is crucial to note that although greater microbiome diversity is generally perceived as advantageous, its impact on CNS functions is still unknown territory and may not universally benefit individuals.

Fecal microbiota transplants from depressed individuals to healthy ones have also been explored in various studies, revealing that rats colonized with microbiota from depressed individuals reproduced depression-like features, demonstrating a link between dysbiotic microbiota and depression. Besides exhibiting depressive behaviors, they also displayed increased plasma kynurenine levels, a metabolite of amino acid tryptophan [126]. A study involving germ-free mice transplanted with fecal microbiota from MDD patients also demonstrated that depressive characteristics can be transmitted through the gut microbiome [128].

Alterations in gut microbiota composition can also exert an influence on serotonin levels, which is recognized for its role in the pathophysiology of depression. Serotonin is produced from its precursor, tryptophan, in the raphe nuclei, enterochromaffin cells, and the enteric nerves within the gut. In particular, around 95% of the body’s total serotonin is produced in the gut, with the majority found in plasma [129]. This neurotransmitter modulates the interaction between the gut and the CNS, mediating intrinsic gut reflexes such as motility and vasodilation [130]. It can also regulate interstitial cells of Cajal through serotonin (5-HT) 5-HT2B receptors, which are specialized mesenchymal cells responsible for electrical slow waves in gastrointestinal muscles [107]. The serotonergic system can also exert control on both innate and adaptive immune cells. Also, 5-HT receptors (5-HTRs) are expressed on various T cells, facilitating their activation and proliferation through 5-HTR3- and 5-HTR1-mediated signaling. Furthermore, serotonin can also serve as a pro-inflammatory mediator and incite gut inflammation by mechanisms that involve increased oxidative stress, activation of redox-sensitive transcription factors, and increased proinflammatory cytokines. For example, 5-HT signaling stimulates M2 macrophage polarization and affects cytokine secretion by macrophages and dendritic cells [130].

The metabolism of tryptophan follows two opposing paths, either leading to the formation of serotonin from 5-hydroxytryptophan or the generation of kynurenine or quinolinic acid. In response to inflammatory processes, the enzyme indoleamine 2,3-dioxygenase is activated, which converts tryptophan into kynurenine. The activation of the kynurenine pathway is part of the immune response to pathogens and injury. Altered kynurenine metabolism can disrupt the balance of serotonin, potentially contributing to depressive states. Indeed, there is a shift in tryptophan metabolism from serotonin to the kynurenine pathway in depressed patients [131]. The kynurenine pathway metabolism is regulated by inflammatory mediators and is influenced by the gut microbiota, which dynamically interacts with the host to regulate the immune system. Germ-free animals exhibit an immature immune system and reduced kynurenine pathway metabolism, which can be normalized by introducing gut microbiota [132].

The levels of serotonin, both in the peripheral system and the brain, can be influenced by the composition of the intestinal microbiota. Some specific genera, such as *Candida*, *Streptococcus*, *Escherichia*, and *Enterococcus*, possess the capability to directly synthesize serotonin. *Bacteroides* species, abundant in patients with MDD [133], can impact the tryptophan availability and in the synthesis of serotonin along the gut–brain axis since these bacteria convert tryptophan to indole and its derivatives [134]. As an example, *B. uniformis* and *B. fragilis* are microbial variants that alone are capable of enhancing susceptibility to stress-induced depression-like behaviors, causing metabolic serotonin disturbances and inhibiting hippocampal neurogenesis in mice [133]. In postpartum women with severe depression, it was described as a dysregulated kynurenine pathway, reduced serotonin levels, increased IL-6 and IL-8 levels, and reduced IL-2 and quinolinic acid concentrations in plasma [135], suggesting a role of the kynurenine pathway along with inflammatory cytokines in this condition. Although still inconclusive, the changes observed in the composition of gut microbiota in humans and rodents with postpartum depression may be an important factor behind the cause of the disease.

### 4.2. The Impact of Prenatal Maternal Stress on Maternal Offspring Microbiome Dynamics

During pregnancy, individuals may experience various stressors, encompassing adverse life events like natural disasters, wars, periods of famine, parental conflict, and financial or relationship stress, alongside experiences of depressive symptoms and/or anxiety. Depression is one of the most prevalent psychiatric disorders during pregnancy, posing serious adverse impacts on both maternal and infant health; it is relevant to emphasize that anxiety and depressive disorders are among the most prevalent psychiatric conditions and often occur together. An analysis of 173 studies on the occurrence of prenatal depression found that the prevalence of any antenatal depression is 20.7%, and the prevalence of major antenatal depression is 15% [136]. Experiencing depression, anxiety, or stress during pregnancy can expose both mother and infant to numerous risks. Psychologically, these include impaired bonding with the fetus and newborn, a higher likelihood of experiencing difficulties in coping with the emotional and psychological demands of the postnatal period, and postnatal depression [137]. Moreover, women suffering from depression during pregnancy are at higher risk of substance abuse [138]. Adverse outcomes in the offspring include premature birth, low birth weight, intrauterine growth restriction [139], and increased risk of psychiatric disorders such as depression and anxiety disorders [140,141], as well as changes in the intestinal microbiota and immune system [142]. In addition to major factors related to prenatal depression, such as a history of depression, marital status (single/separated/divorced), lack of social support, exposure to violence, unemployment, unplanned pregnancy, and history of smoking [136], gut dysbiosis can also play a role. Gut dysbiosis decreases the levels of estrogens and progesterone by altering their synthesis hormones [143], which can affect the mother’s mood.

Emerging studies in both animals and humans indicate a connection between maternal stress and depression with changes in the infant gut microbiota, which, in turn, affect gastrointestinal maturation and immune function. Specifically, maternal stress during pregnancy has been associated with reduced alpha diversity within the infant microbiome—a measure of the variety of species within a microbial community. This reduction in diversity indicates significant changes in the microbial composition of the offspring [144,145,146].

As aforementioned in Section 4.1, extended periods of stress may significantly affect gastrointestinal health by activating the HPA axis, resulting in “leaky gut”. The correlation between prenatal maternal stress and neuropsychiatric disorders in offspring is influenced by various factors, and potential mechanisms linking maternal prenatal cortisol concentrations to the infant intestinal microbiota have been described [147] since the intestinal microbiota is recognized for influencing the development of the HPA system.

Maternal stress can disrupt placental function, altering the fetal environment and increasing the levels of cortisol [148]. This disruption also poses challenges to the coordination between maternal glucocorticoids and the immune system, contributing to heightened maternal inflammation [149,150]. Notably, prenatal stress, even in cases where cortisol levels are not elevated, yielded a comparable impact on the infant microbiota as elevated cortisol in the absence of reported stress [147], suggesting that maternal stress (disturbed sleep or irregular diet) does not necessarily involve changes in the HPA axis, and vice-versa. Mothers experiencing stress or anxiety may transfer an altered microbiota to their infants or expose them to increased cortisol concentrations through placental transfer [147]. Reduced expression of the 11-beta-hydroxysteroid dehydrogenase type 2 gene in the placenta was associated with maternal anxiety in late pregnancy. This enzyme is involved in the conversion of the stress hormone cortisol to its inactive form, cortisone, which can explain alterations in placental function and increased fetal exposure to cortisol following prenatal stress [149].

Prenatal stress is related to alterations in the composition of the maternal gastrointestinal and vaginal microbiota, which have been associated with effects on the gut microbiota of the offspring [146]. Several studies have offered perspectives on how diverse maternal stressors may leave enduring imprints on the microbial landscapes of the next generation. In a study by Zijlmans [147] involving 56 healthy, vaginally born infants, it was found that maternal prenatal stress, measured through self-reports and/or salivary cortisol levels, resulted in lasting changes to the infant’s microbiota. These effects persisted up to 16 weeks of age, with adjustments made for breastfeeding and maternal postnatal stress having no significant impact. The altered microbiota showed a decrease in lactic acid bacteria and Actinobacteria, along with an increase in Proteobacteria like *Escherichia*, *Serratia*, and *Enterobacter*, a group of bacteria known for causing infection. This specific colonization pattern was strongly correlated with maternal reports of gastrointestinal symptoms and allergic reactions in the infant. The suggested mechanisms propose that maternal prenatal cortisol concentrations could cause dysregulation of the fetal HPA axis, resulting in higher cortisol concentration and alterations in the immune cells in the gut, ultimately affecting the composition of the infants’ microbiota.

A study conducted by Galley et al. (2023) [145] unveiled a correlation between maternal anxiety, depression, or perceived stress, and the abundance of various bifidobacterial species in infant stool samples within the first 13 months of life. Bifidobacteria, recognized as early colonizers of the infant gut, displayed potential dysfunction in the colonization process among offspring born to mothers experiencing stress, anxiety, or depression. In a noteworthy study conducted on pregnant women from South Africa, distinct psychological profiles during pregnancy were found to be associated with diverse effects on fecal bacteria. Specifically, exposure to lifelong intimate partner violence emerged as a significant factor linked to variations in both infant and maternal fecal bacteria. Infants born to mothers experiencing intimate partner violence exhibited a high proportion of the Enterobacteriaceae family (genus *Citrobacter* and three unclassified genera) at birth [151].

A recent study involving women who experienced stress during the third trimester of pregnancy found a correlation between prenatal stress and increased microbial diversity in their infants, as measured by stool samples collected at 1 month of age. It is noteworthy to observe that infants exposed to higher stress levels showed a greater abundance of *Lactobacillus* and *Bifidobacterium*, which are generally considered beneficial. Conversely, those exposed to lower stress levels exhibited a higher abundance of *Bacteroides*, *Eggerthella*, and *Enterobacteriacea*, indicating that differences in the abundance of microorganisms can be observed between infants exposed to higher versus lower levels of prenatal stress [152]. Generally, greater gut microbial diversity is seen as beneficial, which is linked to better health and resistance to stressors. For example, duration and frequency of physical activity, or eating more fruits and vegetables, are positively correlated with microbiome diversity [153]. However, while ecological competition generally improves microbiome stability, the high species diversity in the mammalian microbiome can actually destabilize it. According to the model by Coyte et al. (2015) [154], although increasing species numbers tends to destabilize the microbiome, the introduction of competition can create negative feedback loops that have a stabilizing effect. This means that a diverse and competitive microbial community may lead to a stable microbiome despite the destabilizing effects of increased species numbers.

Altered colonization patterns and gut dysbiosis are also evident in preclinical studies utilizing animal models of chronic prenatal stress. Pregnant rats subjected to restraint stress in late gestation had their male offspring assessed for bacterial composition of fecal content at 4 months of age. Notably, prenatal stress caused enduring alterations in the intestinal microbiota of the offspring. Those exposed to prenatal stress exhibited a higher abundance of three genera within the Clostridiales order: *Oscillibacter*, *Anaerotruncus*, and *Peptococcus*. Furthermore, there was a tendency toward reduced bacterial counts in the *Lactobacillus* genus [155]. *Lactobacillus*, a probiotic strain, is known to enhance mucosal barrier function, demonstrating antagonistic effects against intestinal pathogens and offering protection against inflammatory bowel disease [156,157]. Gur et al. (2019) [158] observed that maternal restraint stress may also reduce *Bacteroides* and *Parabacteroides* in male offspring of mice, genera which may be associated with abnormalities in social behaviors [159].

In another study involving rodents, offspring from dams exposed to prenatal stress exhibited a significant imbalance in bacterial abundance. Specifically, there was a reduction in anti-inflammatory probiotics, including Bifidobacteriales, Bifidobacteriaceae, and *Bifidobacterium*. Conversely, this group also displayed an increase in pro-inflammatory bacteria such as Desulfovibrionales, Desulfovibrionaceae, and *Desulfovibrio*. Moreover, the abundance of *Desulfovibrio* was correlated with the level of inflammation in offspring mice, suggesting that its excessive proliferation may contribute to the development of inflammatory diseases in adulthood, such as increasing susceptibility to colitis [160]. Gut dysbiosis was also observed in a study conducted with monkeys, revealing a reduction in both *Lactobacilli* and Bifidobacteria in infants born to females exposed to repeated stress during pregnancy [99].

Despite these discrepancies, evidence suggests that prenatal stress exposure can disrupt the diversity of the intestinal microbiome in both mothers and offspring, potentially affecting the neurodevelopment of the offspring [158]. The gut–brain connection emerges as a critical link between maternal prenatal stress and changes in behaviors, including social and depressive behaviors [158,161,162]. Notably, alterations in specific bacterial genera are correlated with changes in HPA axis reactivity [163]. Moreover, prenatal stress induces exaggerated HPA axis responses to stress, as evidenced by prolonged corticosterone release, elevated blood pressure, and impaired cognitive function in the offspring [155].

Following postnatal microbial colonization, a series of events influences the neural processing of sensory information related to the HPA axis [106], contributing to mood and mental health outcomes in the offspring. Jašarević and Bale’s (2019) [164] review suggests mechanisms through which maternal microbial signals impact prenatal and early postnatal development. During pregnancy, the maternal gut microbiota supplies essential metabolites for fetal growth, driving immune cell expansion, neural circuit formation, and metabolic provisioning. However, maternal exposure to stress may alter maternal gut microbiota composition, function, and availability of microbiota-derived metabolites, exerting effects on fetal growth, brain development, and psychiatric disorders. The diversity and composition of the infant stool microbiome correlate with maternal levels of anxiety, depression, and stress during pregnancy. For instance, bifidobacteria is a significant component of the infant gut microbiota [165]. Changes in infant bifidobacteria have been associated with maternal anxiety and depression. Moreover, inflammation during pregnancy affects the composition of bifidobacteria in their infants [145].

*Bifidobacterium dentium*, a species of bacteria found in the oral and intestinal microbioma, comprises about 0.7% of the microbiome in healthy adults. Mice colonized with *B. dentium* and its metabolites exhibited increased intestinal serotonin levels, higher expression of 5HT2AR, 5HT4R, and the serotonin transporter, and a greater number of serotonin–chromogranin–positive enterochromaffin cells. Moreover, *B. dentium* normalized anxiety-like behaviors of germ-free mice, emphasizing that modulation of the serotoninergic system by a particular microbe may provide new strategies to target depression/anxiety [166]. Thus, imbalances in specific microbial taxa for maintaining optimal immune function may impact offspring health, increasing the vulnerability to immune and psychiatric diseases.

The dysfunction in serotonin production and neurotransmission is proposed as a potential mechanism through which alterations in the microbiome contribute to mental health disorders, including MDD. In addition to the association between *B. dentium* and depressive disorders, it has been reported that the Enterobacteriaceae family, which includes enteric pathogens promoting an inflammatory response, is also linked to depression in an adult population [123]. Furthermore, the prevalence of IgA and IgM against LPS of these bacteria is greater in patients with MDD [167].

Table 1 summarizes the changes induced by prenatal stress exposure in the offspring microbiota and specific outcomes.

### 4.3. The Impact of Maternal Microbiota and Prenatal Stress on Offspring Immune System

Concerning the immune system, the vertical transmission of maternal microbiota during birth also influences postnatal immune development, eliciting a potent immunostimulatory effect in offspring [164]. Maternal microbial signals have the capacity to shape the offspring’s immune response, affecting factors such as innate leukocyte populations, transcriptional profiles, and overall functional outcomes. These effects are mediated by the transfer of microbial compounds and facilitated by maternal antibodies [87]. Moreover, microbial metabolites, particularly short-chain fatty acids (SCFA) produced by the maternal microbiota, play a regulatory role in intestinal immunity, T-cell development, and overall immune function [168]. Findings from mouse models of prenatal stress suggest that alterations in the maternal gut microbiome due to stress can affect the immune system of offspring. Chen et al. (2022) [169] observed a reduction in alpha diversity in fecal samples from stressed dams, and prenatal stress increased circulating levels of neutrophils and CD8 T-cells in the offspring. Another study analyzed mucosal immunity in pups exposed to prenatal stress, revealing a significant decline in the production of intestinal secretory IgA [160]—an antibody class that prevents the adhesion and penetration of pathogens through the intestinal barrier [170]. Additionally, prenatal stress exposure can increase levels of intestinal inflammation as evidenced by increased transcript levels of pro-inflammatory cytokines and the frequency of inflammatory monocytes [160,171]. Prenatal stress exposure reduced the expression of occludin, decreased the number of secretory immunoglobulin A, and increased the expression of IL-1β, IFN-γ, and TNF-α [160].

Understanding these mechanisms offers insights into how stress during pregnancy may affect the early development and programming of the offspring’s immune system, potentially increasing vulnerability to mental health disorders later in life.

## 5. Ethanol-Induced Changes in Gut Microbiota: Understanding Consequences and Investigating Prenatal Exposure Effects

### 5.1. Consequences of Ethanol Consumption in Intestinal Dysbiosis

Alcohol is a widely accepted psychoactive substance in society, despite its addictive properties. Alcohol misuse globally stands as the seventh-leading risk factor for premature death and disabilities [172]. In 2018, the World Health Organization (WHO) reported that alcohol contributes to over 200 diseases and injury-related health conditions, encompassing liver diseases, cancer, cardiovascular diseases, tuberculosis, road injuries, violence, suicides, and HIV/AIDS [173].

Gastrointestinal (GI) diseases are common among those who consume alcohol excessively. The direct contact of alcohol with the mucosa that coats the upper gastrointestinal tract may induce various metabolic and functional changes, leading to significant mucosal damage that can result in gastritis, GI bleeding, and diarrhea. Chronic alcohol misuse is also linked to cancers of the digestive tract, including those of the oral cavity, esophagus, liver, colorectum, stomach, bowel, and pancreas [174].

Alcohol increases intestinal permeability, with decreased expression of occludins, and induces mucosal injuries in the gut, allowing the translocation of large molecules like endotoxins (i.e., LPS) and other bacterial toxins from the gut to the blood or lymph [175]. These toxic substances may cause harmful effects on multiple organs. The LPS binds to TLRs on immune cells, activating inflammatory cytokines. This process explains the elevated markers of monocyte activation and heightened immune response observed in excessive drinkers, particularly those with recent alcohol intake [176]. The ongoing communication between the gut microbiome and the brain facilitates the induction of neuroinflammation by cytokines, which is associated with the development of neuropsychiatric disorders [93].

Alcohol produces an imbalance in the intestinal bacterial population, generating intestinal dysbiosis [177,178], evidenced in both clinical data [179,180,181,182,183,184] and preclinical experiments [12,185,186,187,188]. Alcohol consumption correlates with reduced levels of commensal bacteria and probiotic species such as *Lactobacillus* and *Bifidobacterium*, along with important physicobiotics such as *Faecalibacterium prausnitzii* and *Akkermansia muciniphila* [178], and also *Roseburia, Blautia*, *Bacteroides*, Lachnospiraceae, and *Enterococci* [180]. On the other hand, Firmicutes is the phylum that has the greatest proliferation in the presence of alcohol, and genera *Adlercreutzia, Allobaculum*, and *Turicibacter* have been associated with anxiety- and depression-like disorders [188,189]. Proteobacteria and many species of Bacteroidetes also proliferate in the presence of alcohol [179]. Proteobacteria are a phylum that hosts many known bacteria such as *Brucella, Rickettsia, Bordetella, Neisseria, Escherichia, Shigella, Salmonella, Yersina*, and *Helicobacter*. Proteobacteria commonly exhibit Gram-negative staining, indicating the presence of LPS in their outer membrane. They are frequently elevated in disease conditions and have been suggested as a potential marker of microbiota instability [190]. Cuesta et al. (2021) [191] demonstrated a reduction in Gram-positive bacteria taxa, specifically a decrease in the phylum Firmicutes, possibly due to a decrease in Lachnospiraceae family, in mice treated with alcohol for 3 months. Additionally, the Muribaculaceae family and Alloprevotella genus increased in C57BL/6J mice after alcohol consumption. These findings are consistent with higher intestinal permeability induced by chronic ethanol, which may lead to inflammation.

The harmful effects of ethanol may arise directly from its toxicity or indirectly through its metabolites and the generation of reactive oxygen species (ROS) [192]. ROS are chemically reactive molecules containing oxygen, which can cause damage to cells and tissues. The production of ROS during ethanol metabolism can create an environment that is hostile to anaerobic bacteria, promoting the enrichment of aerotolerant bacteria [175,191,193].

Alcohol Use Disorder (AUD) is a chronic and relapsing condition characterized by an inability to control or reduce alcohol consumption [172,173]. Individuals with AUD who had high intestinal permeability showed alterations in their gut microbiota composition, such as a decrease in overall bacterial abundance and in specific bacterial families like Ruminococcaceae (*Ruminococcus*, *Faecalibacterium*, *Subdoligranulum*, *Oscillibacter*, and *Anaerofilum*), along with an increase in the abundance of Lachnospiraceae (genus *Dorea*) and the genus *Blautia* [181]. Bacteria from the Ruminococcaceae family, which increase during alcohol abstinence, are known to have a beneficial impact on intestinal barrier function [194]. *Faecalibacterium prausnitzii* is a bacterial species known for its anti-inflammatory properties, and its reduction is related to Crohn’s disease and ulcerative colitis [195,196]. The Lachnospiraceae family is a butyrate-producer. Butyrate, a SCFA produced by symbiotic bacteria in the GI tract through the fermentation of dietary fibers, has functions that include strengthening the gut barrier, reshaping the gut microenvironment, and suppressing inflammation [197,198]. This substance has a protective effect on the intestinal barrier as it leads to a reduction in intestinal permeability induced by alcohol [199]. The lower availability of butyrate is associated with increased intestinal permeability [200]. In the study by Singhal et al. (2021) [201], mice fed with a Lieber–DeCarli alcohol liquid diet exhibited a reduction in the abundance of the butyrate-producing family Lachnospiraceae, in contrast to the findings with AUD individuals found in Leclercq et al. (2020) [202].

Mice transplanted with feces from patients with AUD showed a decrease in *F. prausnitzii* and an increase in Lachnospiraceae *abundance* [201]. Since *F. prausnitzii* is positively correlated with butyric acid levels, which are protective against inflammation [203], this reduction suggests a potential link between gut microbiota alterations and inflammatory processes in AUD. Furthermore, these mice exhibited behavior similar to depression, including reduced sociability and decreased lipolysis [204].

Depressive behavior induced by chronic alcohol consumption was attenuated by dietary administration of sodium butyrate in mice. This compound reduced inflammation and suppressed microglia-mediated neuroinflammation through the GPR109A/PPAR-γ/TLR4-NF-κB-signaling pathway, a complex signaling cascade involved in regulating inflammation and immune responses. Moreover, sodium butyrate ameliorates gut dysbiosis caused by chronic alcohol exposure by increasing the abundance of beneficial bacteria such as *Faecalibaculum* and *Alloprevotella* [198].

Intestinal dysbiosis caused by alcohol has been associated with learning and memory dysfunction, depression, anxiety, and alcohol craving [161,202,204]. Additionally, it has been implicated in perpetuating and promoting drinking behavior [205]. In mice, exposure to alcohol for 3 weeks showed an increase in *Adlercreutzia*, *Allobaculum*, and *Turicibacter*, which are bacteria related to intestinal inflammation [206] and neuroimmune regulation [207]. It is noteworthy that *Adlercreutzia* was negatively correlated with anxiety-like behavior, positively with alcohol preference, and negatively with the expression of brain-derived neurotrophic factor (BDNF) and Gabra1 in the prefrontal cortex [188]. Since BDNF deficiency is associated with an increased preference for alcohol [208] and Gabra1 deficiency leads to alcohol-related cerebral hyperexcitability [209], these correlations suggest that *Adlercreutzia* may influence alcohol preference and brain excitability through its impact on BDNF and Gabra1 expression.

Probiotics administration, specifically *Lactobacillus rhamnosus*, *Lactobacillus acidophilus*, and *Bifidobacterium*, protected mice from chronic ethanol exposure-induced depressive-like behavior and hippocampal neuronal damage. Changes in the hippocampus are significant in depression, as reduced hippocampal volume is a clinical depression marker [210,211]. *Bifidobacterium*, the most widely used probiotic, seems to have positive effects in alleviating the symptoms of depression [212]. *Bifidobacterium infantis* has been linked to an increase in the serotonin precursor tryptophan [178]. Although tryptophan can generate serotonin, the main pathway of metabolism is the kynurenine pathway, where tryptophan is transformed into kynurenine. An increase in kynurenine levels after chronic intermittent alcohol consumption has been revealed, both in the periphery and in the brain. Moreover, this regimen of alcohol exposure induced anhedonia, which was prevented by antibiotic treatment [213].

Exposure to ethanol increases plasma LPS permeability and activates NF-kB, leading Kupffer cells to produce pro-inflammatory chemokines like TNF-α and cytokines [214]. It also raises levels of inflammasome components such as NLRP1, NLRP3, and ASC [215]. Using AAV transfection to downregulate hippocampal NLRP3 expression, Yao et al. (2023) [211] demonstrated that this downregulation decreased depressive behavior in animals exposed to chronic alcohol consumption, suggesting that gut microbiota can regulate depressive behavior through NLRP3-mediated hippocampal neuroinflammation. Additionally, they found that serum inflammatory cytokines mediate the link between gut microbiota and depressive behavior in these mice. Chronic alcohol consumption increased inflammatory markers (IL-1β, iNOS, TNF-α, COX-2, IL-10, and CXCL10) in the colons of wild-type mice but not in TLR4-KO mice. The TLR4-KO mice also exhibited a distinct microbiota, characterized by a reduced abundance of Firmicutes, potentially offering protection against ethanol-induced inflammation [191]. Moreover, TLR4 exerts an important role in alcohol-induced neuroinflammation [216] and depression [217], underscoring the significance of neuroinflammation in these disorders.

These findings highlight the critical role of the gut microbiota in mediating the relationship between alcohol abuse, immune system activation, and depressive behavior. The observed protective effect in TLR4-KO mice, with their distinct microbiota composition, suggests that targeting the microbiota–immune system axis could be a promising strategy for mitigating the negative effects of chronic alcohol consumption.

Table 2 summarizes the relationship between alcohol exposure, microbiota dysbiosis, and associated outcomes.

The exact mechanisms of alcohol-induced intestinal dysbiosis have not yet been elucidated. However, there are a series of hypotheses, such as slow peristalsis, increased fecal pH [175,218], and changes in bile salt concentrations [219]. Changes in luminal pH can potentially shift the competitive balance among different bacterial communities [175]. Dysbiosis and altered bile acid pools are linked to liver diseases like cirrhosis and metabolic syndrome. With advancing liver disease, dysbiosis occurs alongside decreased intestinal bile acid concentration, favoring Firmicutes and increasing deoxycholic acid production. Alcohol intake exacerbates these effects, leading to greater intestinal and systemic inflammation and worsening dysbiosis [219]. Due to the role of the gut microbiome in the gut–brain axis and its influence on AUD, manipulating the microbiota offers a promising therapeutic approach for AUD treatment.

### 5.2. Effects of Prenatal Ethanol Exposure on Maternal and Offspring Microbiota

Alcohol consumption during pregnancy may pose significant health risks for women and their offspring [220,221]. A household survey conducted by Tan et al. (2015) [222] found that 10.2% of pregnant women reported any alcohol use, with 3.1% engaging in binge drinking. Additionally, 1 in 10 women reported alcohol use in the past 30 days, and 1 in 33 reported binge drinking during pregnancy. Considering the aforementioned, there is no safe dose of alcohol during pregnancy [223] as prenatal alcohol exposure (PAE) has been linked to adverse behavioral effects [224], as well as learning and memory impairments [225,226]. PAE is also associated with neurological disorders [227], involving multiple mechanisms that contribute to developmental harm and adverse outcomes [5]. Although the impact of PAE on the intestinal microbiota has been relatively understudied, some studies have shown significant consequences in both offspring and mothers.

In a study involving pregnant women who consumed alcohol, fecal samples were collected at the end of pregnancy and from their newborns within 48 h after birth. These samples were compared with those from a non-alcohol consumption group. This comparison revealed a significant change in the maternal gut microbiota, characterized by a reduced abundance of the *Faecalibacterium* genus and an increased abundance of the *Phascolarctobacterium* and *Blautia* genera in the group of women who consumed alcohol. Additionally, the colonization of the offspring’s gut microbiota was impacted, indicating an overgrowth of *Megamonas* [15]. There is a predominance of certain bacterial genera, including *Megamonas*, in fecal samples from patients diagnosed with MDD, suggesting that an increased presence of this genus could be associated with this disorder [123]. Indeed, it is known that children exposed to alcohol in utero may be predisposed to exhibit higher levels of negative emotionality, thus presenting an increased risk for childhood depression [228].

PAE appears to have a lasting impact on the fecal microbiota of offspring, as revealed in a study involving rats that exhibited changes in bacterial diversity throughout adulthood. The genera *Bacteroides*, *Roseburia*, and *Proteus* are the most abundant in PAE animals. The authors also conducted analyses with stratification by sex and observed differences in the microbiota between males and females. Notably, males exhibited a higher α diversity, whereas no significant differences were discerned in females. Furthermore, variations in different genera were identified based on sex. Among males, the genus *Akkermansia* was detected in higher abundance, while *Bifidobacterium* displayed a lower abundance. Conversely, in females, there was an elevated abundance of the genera *Proteus*, *Roseburia*, and *Faecalitalea*. In both sexes, the genus Bacteroides exhibited higher abundance, while Ruminococcus displayed lower abundance in cases of PAE [229]. Some of these altered bacterial genera are associated with inflammatory conditions, such as irritable bowel syndrome. Studies have demonstrated that individuals with irritable bowel syndrome experience a reduction in *Bifidobacterium* and an increase in *Bacteroides* [230,231]. Furthermore, besides *Bifidobacterium*, bacteria such as *Roseburia* and *Akkermansia* also play a role in regulating the immune system by producing SCFA, which are important metabolites of the gut microbiome [210,232,233]. Levels of SCFAs, such as acetic acid, propionic acid, and pentanoic acid, have been shown to be reduced in animal models of depression [234].

Virdee et al. (2021) [235] revealed a distinct and significant biosignature of microbe-dependent products (MDPs), which distinguishes alcohol-exposed mothers from their controls. Maternal plasma was characterized by phenolic acids, which are associated with anti-inflammatory actions through the inhibition of pro-oxidant enzyme-signaling cascades [236]. Probably, their elevation in PAE may potentially mitigate some of the damage generated by alcohol in both the mother and the fetus. Many metabolites from maternal plasma are readily exchanged across the placenta and become bioavailable to the fetus [235]. Several MDPs elevated by PAE (catechol sulfate, 4-ethylphenylsulfate, erythritol, indolepropionate, oxindole, p-cresol, salicylate) are related to neuroinflammation, depression, anxiety, and autism [235].

Analysis of fecal samples from pregnant dams with *ad libitum* access to a liquid diet, containing alcohol with 36% of total calories derived from ethanol, showed lower bacterial diversity and uniformity, as well as a general increase in aerotolerant bacteria Desulfovibrionaceae and *Akkermansia* (Verrucomicrobiota) compared to the control group. The offspring of these dams exposed to ethanol showed an increased abundance of *Parabacteroides*, *Alistipes*, and the phylum Firmicutes, as well as a decrease in *Ruminococcus* [17].

Ethanol may lead to alterations in gut microbiota composition, influencing cytokine production and release. These cytokines, in response to changes in the gut microbiota, may contribute to inflammation and immune system dysregulation, potentially exacerbating the effects of ethanol exposure on various physiological processes, including depression, anxiety, and alcohol dependence [237].

Alterations in cytokine levels due to maternal alcohol exposure can influence neurocognitive and neuropsychiatric outcomes in children by affecting inflammatory components [230,238]. Increased levels of IL-15 and IL-10 were observed in the maternal blood samples collected during the second trimester in alcohol-consuming mothers, which were linked to neurodevelopmental delays in their offspring [228]. These findings suggest that maternal immune system dysregulation may contribute to adverse child outcomes and could serve as early biomarkers for neurodevelopmental delay, including alcohol-related conditions [228]. Pascual et al. (2017) [238] demonstrated maternal and fetal immune responses triggered by alcohol consumption during pregnancy, which was demonstrated by cytokines/chemokines’ production in fetal and postnatal brains, with upregulation of IL-1β, macrophage inflammatory proteins (MIP)-1α, and fractalkine in cortices of 15-day-old fetuses. Bake et al. (2021) [239] found sex-dependent effects on cytokine levels of prenatal alcohol exposure using pregnant rats exposed to ethanol vapor. They found that males exhibited suppressed cytokine levels and increased anxiety-like behavior, whereas females displayed elevated cytokine levels in adipose tissue and liver along with decreased social interaction. All these alterations could, at least in part, result from changes in intestinal barrier/permeability and microbiota.

Table 3 summarizes the changes induced by prenatal alcohol exposure in the mother and offspring microbiota.

## 6. Connecting the Dots: Exploring the Link between Microbiota Dysbiosis, Maternal Immunological Changes, and Depression

The microbiome encompasses the entire collection of genomes from all microorganisms within a particular environment, which includes the community of microorganisms, their products, and the environmental conditions they inhabit. The microbiome plays a crucial role in the overall function and effectiveness of both the innate and adaptive branches of the immune system, while the immune system helps maintain key aspects of its symbiotic relationship with microbes [18]. Imbalances in these interactions may contribute to the development of various immune-mediated disorders. High rates of depression have been observed in various conditions involving immune system activation [101].

The regulation of the maternal immune system throughout pregnancy is a complex and finely tuned process designed to protect the developing fetus. This regulation involves suppressing certain immune activities in order to create a favorable environment for fetal growth. Disruptions in the maternal immune system may cause significant consequences, particularly regarding the production of cytokines. Notably, studies indicate that cytokines such as IL-6, IL-1α, and TNF-α can move bidirectionally across the placenta, illustrating a dynamic transfer of these molecules during pregnancy [240]. Therefore, changes in the maternal microbiota may contribute to immune dysregulation, influencing subsequent effects on fetal development.

**Figure 2:**
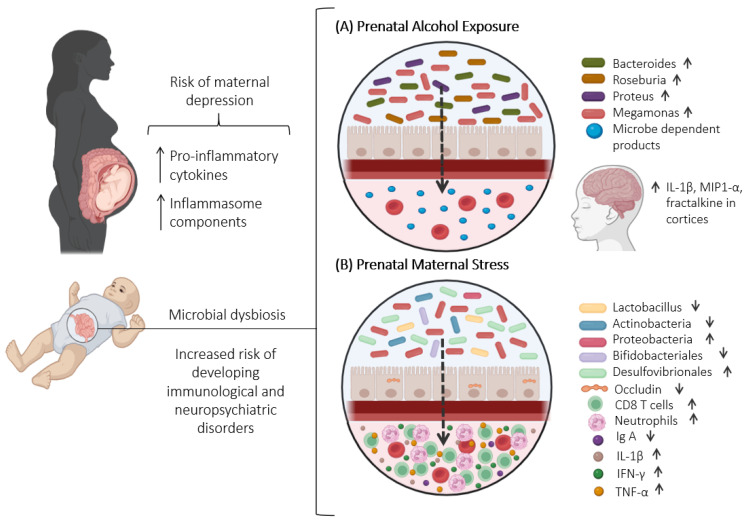
Prenatal alcohol/stress alters the microbiota and immune function of the offspring. Exposure to prenatal ethanol or stress may induce alteration in cytokines levels, shifting them towards a proinflammatory state. Inflammation during pregnancy can lead to lasting changes in both innate and adaptive immune functions in newborns, increasing the risk of developing depression. ↑ = upregulated; ↓ = downregulated.

Cytokine transfer through the placenta is vital for proper fetal development, creating a direct communication link between the maternal immune system and the developing fetus. Disruptions in this delicate balance might result in imbalances in cytokine levels, potentially affecting the fetal immune environment with consequences extending beyond the prenatal period. Exposure to prenatal ethanol or stress has been shown to cause alterations in cytokine levels towards a proinflammatory state. Important exposure to inflammation in pregnant mothers led to lasting changes in both innate and adaptive immune functions in their newborns [241], potentially contributing to the development of depression later in life [242]. This fact has particular significance in the realm of neurodevelopmental disorders, where inappropriate activation of the maternal immune system has been identified as a contributing factor to psychosis, depressive- and anxiety-like behaviors, and increased substance use behavior [243,244,245] (Figure 2).

The microbiota–gut–brain axis emerges as a key system in regulating various CNS homeostatic processes, including neurotransmission, activation of stress responses, and neuroinflammation [246,247,248]. Alterations in immune function, such as an increased production of pro-inflammatory cytokines, have been observed in psychiatric conditions like depression and anxiety. The bidirectional communication between the immune system and the CNS is a complex interplay that influences both immune responses and brain function. Chronic inflammation, triggered by persistent immune activation, can affect neurotransmitter metabolism, neuroendocrine function, and neural circuits related to mood and behavior. This interaction highlights the intricate relationship between the immune system and psychiatric health, suggesting that immune dysregulation may contribute to the development and exacerbation of psychiatric disorders. This gains particular interest when the immune alterations are in the mother with consequences in the offspring.

## 7. Conclusions

Based on the reviewed studies, it can be concluded that maternal exposure to stress and ethanol significantly affects the microbiota of both the mother and her offspring through multiple interconnected mechanisms. Stress and ethanol activate the HPA axis, leading to increased levels of corticosterone in rodents and cortisol in humans. Elevated glucocorticoids disrupt gut permeability, resulting in a “leaky gut” and enhanced translocation of LPS into the bloodstream. The activation of inflammasome components contributes to systemic inflammation and alterations in gut microbiota composition. Dysbiosis during pregnancy can negatively impact the maternal–fetal interface, further influencing the immune system function. These disruptions can impair the offspring’s immune system and increase their susceptibility to psychiatric disorders later in life. Therefore, it is essential not to underestimate the effects of minor stressors or moderate alcohol intake during pregnancy, as both can significantly contribute to dysbiosis and immune dysregulation, which, when combined, may exacerbate adverse outcomes more than either factor alone. This review highlights the potential cumulative effects of prenatal alcohol and stress exposure on gut microbiota, the immune system, and overall offspring health.

### Implications and Future Research Directions

The findings underscore the need for further research into the combined effects of stress and ethanol on the gut–brain axis. Future studies should explore pharmacological strategies targeting the gut–brain axis as potential interventions to mitigate the effects of maternal stress and ethanol exposure. Additionally, research should investigate the long-term consequences of these exposures on offspring mental health and explore potential therapeutic approaches to address microbiota and immune dysregulation. 

## Figures and Tables

**Table 1:** Prenatal maternal stress and offspring microbiota.

Prenatal Maternal Stress and Microbiota				
Author	Prenatal Stress Exposure	Specie	Microbiota (Offspring)	Outcomes
Zijlmans et al. (2015) doi: 10.1016/j.psyneuen.2015.01.006 [147]	Maternal prenatal stress (self-reports and/or salivary cortisol levels)	Humans	↓ Lactic acid bacteria (*Lactobacillus*, *Lactoccus*, *Aerococcus*); ↓ Actinobacteria; ↑ Proteobacteria (*Escherichia, Serratia* and *Enterobacter*).	Specific colonization patterns were correlated with maternal-reported gastrointestinal symptoms and allergic reactions in the infant.
Galley et al. (2023) doi: 10.1016/j.bbi.2022.10.005 [145]	Maternal anxiety, depression, or perceived stress	Humans	↓ *Bifidobacterium dentium* in the offspring of mothers with higher anxiety, perceived stress, and depression.	*B. dentium* was positively associated with elevated IL-6 and IL-8 during pregnancy
Naudé et al. (2020) doi: 10.1017/neu.2019.43 [151]	Prenatal distinct psychological profiles, including exposure to lifelong intimate partner violence (IPV)	Humans	Infants born to mothers that were exposed to high levels of IPV: ↑ Citrobacter and three unclassified genera (Enterobacteriaceae family) detected at birth.	-
Weiss, Hamidi (2023) doi: 10.1080/14767058.2023.2214835 [152]	Stress during the third trimester of pregnancy	Humans	Lower stress levels: ↑ *Bacteroides*, *Eggerthella*, and *Enterobacteriacea.* Higher stress levels: ↑ *Lactobacillus* and *Bifidobacterium*.	-
Golubeva et al. (2015) doi: 10.1016/j.psyneuen.2015.06.002 [155]	Restraint stress (gestational day 14–20)	Rats	↑ *Oscillibacter*, *Anaerotruncus*, and *Peptococcus*; ↓ *Lactobacillus* (tendency). *male offspring*	-
Gur et al. (2019) doi: 10.1016/j.bbr.2018.06.025 [158]	Restraint stress (gestational day 10–16)	Mice	↓ *Bacteroides* and *Parabacteroides.* *male offspring*	-
Sun et al. (2021) doi: 10.3389/fimmu.2021.700995 [160]	Variable stress beginning at gestational day 10	Mice	↓ anti-inflammatory probiotics: Bifidobacteriales, Bifidobacteriaceae, and *Bifidobacterium*. ↑ pro-inflammatory bacteria: Desulfovibrionales, Desulfovibrionaceae, and *Desulfovibrio*.	Prenatal maternal stress caused persistent overgrowth of *Desulfovibrio* resulting in exacerbated experimental colitis in adulthood.
Bailey, Lubach, Coe (2004) doi: 10.1097/00005176-200404000-00009 [99]	Stressed during pregnancy using an acoustical startle paradigm	Monkeys	↓ *Bifidobacteria* and *Lactobacilli*.	-

↑ = upregulated; ↓ = downregulated.

**Table 2:** Alcohol, microbiota dysbiosis, and outcomes.

Alcohol and Microbiota				
Author	Alcohol Exposure	Specie	Microbiota	Outcomes/Main Results
Mutlu et al. (2009) doi: 10.1111/j.1530-0277.2009.01022.x [185]	Received alcohol or dextrose intragastrically by gavage twice daily for up to 10 weeks	Rats	Alterations in the composition of mucosa-associated microbiota in the colon	Little or no dysbiosis after 4–6 weeks of alcohol feeding, but dysbiosis after 10 weeks of alcohol exposure
Bull-Otterson et al. (2013) doi: 10.1371/journal. pone.0053028 [175]	Mice were fed liquid Lieber–DeCarli diet without or with alcohol (5% *v/v*) for 6 weeks	Mice	↑ Proteobacteria, Actinobacteria ↓ Bacteriodetes, Firmicutes	↑ In plasma endotoxin ↑ Fecal pH ↑ Hepatic inflammation
Leclercq et al. (2014) doi: 10.1073/pnas. 1415174111 [181]	Individuals with AUD	Humans	↑ Lachnospiraceae (*Dorea*) and *Blautia* ↓ Ruminococcaceae (*Ruminococcus*, *Faecalibacterium*, *Subdoligranulum*, *Oscillibacter* and *Anaerofilum*)	↑ Intestinal permeability (IP) ↑ Inflammatory markers (TNFα, IL-1β, IL-6, IL-8, and IL-10) AUD individuals with high IP presented higher depression, anxiety, and alcohol-craving scores
Tsuruya et al. (2016) doi: 10.1038/srep27923 [193]	Individuals with AUD	Humans	↑ *Streptococcus* and *Coprobacillus* ↓ *Bacteroides* and *Ruminococcus* (anaerobes)	Lower production of acetaldehyde from ethanol metabolism
Peterson et al. (2017) doi: 10.1016/j.bbr.2017.01.049 [177]	C57BL/6J mice were exposed to 4 weeks of vaporized ethanol	Mice	↑ *Alistipes* ↓ *Clostridium* IV and XIVb, *Dorea* and *Coprococcus*	↓ α-diversity of microbiota
Dubinkina et al. (2017) doi: 10.1186/s40168-017-0359-2 [180]	Individuals with AUD	Humans	↑ Enterobacteriaceae, ↓ Clostridiales—Individuals with AUD without liver cirrhosis.	Strong negative influence of alcohol dependence and associated liver dysfunction on the intestinal microbiota
Grander et al. (2018) doi: 10.1136/gutjnl-2016-313432 [182]	Individuals with acute alcoholic steatohepatitis	Humans	↓ *Akkermansia muciniphila*	Oral administration of *A. muciniphila* protected against ethanol-induced hepatic injuries
Wang et al. (2018) doi: 10.3389/fmicb. 2018.01874 [187]	Consumption of increasing alcohol concentration 3%, 6%, 10% (*v/v*)	Mice	↑ Firmicutes, Clostridiales and Lachnospiraceae, *Alistipes*, *Odoribacter*	Groups exposed to alcohol or with alcohol abstinence presented anxiety and depression compared
Xiao et al. (2018) doi: 10.1016/j.toxlet. 2018.01.021 [189]	Chronically fed alcohol	Mice	↑ Erysipelotriquia, Erythrobacter, *Allobaculum* and *Blautia*	Increased gut microbiome diversity, with reduced commensal gut taxa. Anxiety behaviors associated with alcohol withdrawal
Xu et al. (2019) doi: 10.1002/biof.1469 [188]	Exposure to 2–8% (*v/v*) ethanol was 21 days	Mice	↑ Actinobacteria and Cyanobacteria ↑ *Adlercreutzia* spp., *Allobaculum* spp., *Turicibacter* spp. ↓ *Helicobacter* spp.	Animals exhibited anxiety/depression-like behaviors
Leclercq et al. (2020) doi: 10.1016/j.celrep. 2020.108238 [202]	Individuals with alcohol dependence	Humans	↑ Lachnospiraceae ↓ *Faecalibacterium praustnizii*	↑ Intestinal Permeability ↑ Introversion and social anxiety
	Microbial transfer from human donors with alcohol dependence to mice	Mice	↑ Firmicutes and *Akkermansia muciniphila* ↓ *Blautia*, *Faecalibacterium*, and Bacteroidetes	↑ Depressive-like behavior ↑ Corticosterone level ↓ Social behavior
Cuesta et al. (2021) doi: 10.3390/ijms222312830 [191]	TLR4 knockout mice treated with 10% (*v/v*) alcohol for 3 months	Mice	↓ Firmicutes	TLR4 is a key factor in determining the intestinal microbiota as TLR4 KO mice did not show the usual signs of ethanol-induced inflammation
Singhal et al. (2021) doi: 10.1080/19490976.2021.1946367 [201]	C57BL/6 mice were fed a Lieber-DeCarli liquid diet containing ethanol 5%(*v*/*v*) for 7 weeks	Mice	↓ Firmicutes, Lachnospiraceace, Clostridiaceae, Ruminococccaceae	Loss of butyrate-producing bacteria and butyrate genes
Yang et al. (2021) doi: 10.1128/AEM.00834-21 [186]	6% (*v/v*) alcohol was added to drinking water for 5 weeks	Rats	↑ Firmicutes and Bacteroidetes ↓ Prevotellaceae, *Ruminococcus-1*, Lachnospiraceae, *Roseburia*, Prevotellaceae and *Lachnoclostridium*	↓ Amino acids and lipids (serum metabolome disorders) ↓ Concentration of serotonin in the hippocampus ↓ Lipopolysaccharide ↑ Intestinal permeability biomarkers
Du et al. (2022) doi: 10.3389/fpsyt. 2022.1054685 [183]	Individuals with alcohol dependence	Humans	↑ *Faecalibacterium*, *Gemmiger* and *Lachnospiracea incertae sedis* ↓ *Megamonas* and *Escherichia*	The cognitive capacity of the participants was assessed using the Montreal Cognitive Assessment (MoCA) and the Mini-Mental State Examination (MMSE), revealing a negative correlation between the MoCA and MMSE scores with years of alcohol consumption and dependence on alcohol.
Baltazar-Díaz et al. (2022) doi: 10.3390/microorganisms10061231 [184]	Individuals with alcoholic cirrhosis	Humans	↑ *Escherichia/Shigella* e *Prevotella* ↓ *Blautia, Faecalibacterium*	↓ α—diversity of microbiota ↓ Acetyl-CoA fermentation to butyrate
Wang et al. (2023) doi: 10.1128/mbio.02392-23 [12]	Individuals with alcohol dependence	Humans	↑ Saccharimonadaceae, Lachnospiraceae, and *Fusobacterium* ↓ Ruminococcaceae, Erysipelotrichaceae, and *Roseburia*	↑ Anxiety and depression behaviors ↑ Preference for alcohol
	Microbial transfer from human donors with alcohol dependence to rats	Rats		
Wei et al. (2023) doi: 10.1016/j.biopha. 2023.114308 [198]	C57BL/6 J mice were fed Lieber-DeCarli liquid diets (28% ethanol) for 6 weeks	Mice	↑ *Streptococcus, Parasutterella, Dubosiella, Muribaculum, Bifidobacterium, Bacteroides, Odoribacter, Alistipes, Lactobacillus* and *Parabacteroides* ↓ *Ileibacterium*, *Lachnoclostridium*, and Coriobacteriaceae	Neuronal necrosis in the hippocampus and prefrontal cortex Exacerbate neurodegeneration and cognitive dysfunction ↑ TNF-α, IL-6, IL-1β and IL-18 in the hippocampus
Yao et al. (2023) doi: 10.1038/s41380-022-01841-y [211]	C57BL/6N mice were exposed to ethanol-free drinking model for 90 days (Fecal microbiota transplantation model)	Mice	↑ Firmicutes, Actinobacteria, Erysipelotrichi, Erysipelotrichales, Bacillales, Erysipelotrichaceae, Staphylococcaceae, *Allobaculum* ↓ Bacteroidetes, Bacteroidia, Verrucomicrobiae	↑ Intestinal permeability ↑ Serum inflammatory factor levels Activation of NLRP3 inflammasome in the hippocampus, leading to neuroinflammation and depressive-like behavior
Carbia et al. (2023) doi: 10.1016/j.ebiom.2023.104442 [179]	Young people aged 18–25 years reported their alcohol use (at least 60 g or more of pure alcohol on at least one occasion in the last 30 days)	Humans	↑ *Veillonella dispar* ↓ *Alistipes*	Difficulties in emotional recognition

↑ = upregulated; ↓ = downregulated.

**Table 3:** Prenatal maternal alcohol and mother/offspring microbiota.

Prenatal Alcohol Exposure and Microbiota			
Author	Alcohol Exposure	Specie	Microbiota/Microbe-Dependent Products
Virdee et al. (2021) doi: 10.1038/s41598-020-80093-8 [235]	Pregnant C57BL/6 mice were gavaged with 3 g/kg alcohol during GD8.5-17.5	Mice	Specific microbe-dependent products (MDP) in the microbiome might serve as a biosignature for identifying PAE Mothers: High levels of MDPs in plasma, most of them phenolic acids and indole derivatives Offspring: High levels of MDPs implicated in neuroinflammation, depression, and anxiety (4-ethylphenylsulfate, oxindole, indolepropionate, p-cresol sulfate, and salicylate)
Wang et al. (2021) doi: 10.3390/biom11030369 [15]	Maternal alcohol consumption during pregnancy	Humans	Mothers: ↑ *Phascolarctobacterium* and *Blautia* ↓ *Faecalibacterium* Offspring: ↑ *Megamonas*
Bodnar et al. (2022) doi: 10.1111/acer.14784 [229]	Pregnant Sprague-Dawley rats received an ethanol diet during GD17-GD21	Rats	Offspring: ↑ *Bacteroides, Roseburia* (at the phylum Firmicutes), and *Proteus* PAE generated sex-specific changes in the microbiota. In males, α-diversity was higher in PAE, and in females, this difference was not detected. Significant alterations in *Bacteroides*, *Bifidobacterium*, *Akkermansia*, and *Ruminiclostridium* were found between PAE and control males. In females, *Bacteroides*, *Proteobacteria*, *Faecalitalea*, *Proteus*, *Roseburia*, and *Gastranaerophilales* were identified to be altered by PAE.
Bodnar et al. (2024) doi: 10.1038/s41598-024-64313-z [17]	Pregnant Sprague-Dawley rats with ad libitum access to an alcohol-containing liquid diet with 36% of total calories derived from ethanol during GD1-GD21	Rats	Mothers: ↑ Desulfovibrionaceae, *Akkermansia* Offspring: ↑ Firmicutes, *Parabacterteroides*, *Alistipes* ↓ *Ruminococcus*

↑ = upregulated; ↓ = downregulated.

## Data Availability

No new data were created or analyzed in this study. Data sharing is not applicable to this article.

## Abbreviations

AUD: Alcohol use disorder; BDNF: Brain-derived neurotrophic factor; CCR9: C-C motif chemokine receptor 9; CNS: Central nervous system; CUMS: Chronic unpredictable mild stress; DC: Dendritic cells; GI: Gastrointestinal; HPA: Hypothalamic-pituitary-adrenal; 5-HT: Serotonin; 5-HTRs: 5-HT receptors; IL: Interleukin; Ig: Immunoglobulin; LPS: Lipopolysaccharides; NF-KB: Nuclear factor kappa B; NLRP3: NOD-like receptor family, pyrin domain containing; MDPs: Microbe-dependent products; MDD: Major depressive disorder; MUC: Mucin; PAE: Prenatal alcohol exposure; ROS: Reactive oxygen species; SCFA: Short-chain fatty acids; Th: Helper T cells; Treg: Regulatory T cells; TGF: Transforming growth factor; TNF-α: Tumor necrosis factor-α; TLR4-KO: Toll-like receptor 4 knockout.
